# Atypical manifestations in CAPS sydrome: not so unfrequent?

**DOI:** 10.1186/1546-0096-13-S1-P66

**Published:** 2015-09-28

**Authors:** S Buján-Rivas, M Basagaña-Torrentó, C Modesto-Caballero, JI Aróstegui-Gorospe, M Vilardell-Tarrès, J Yagüe

**Affiliations:** 1 Hospital Vall D'Hebron, Internal Medicine, Barcelona, Spain; 2 Hospital Germans Trias I Pujol, Allergology, Barcelona, Spain; 3 Hospital Clinic I Provincial, Immunology, Barcelona, Spain

## 

Clinical picture of CAPS syndrome includes periodic fever, skin rash, arthritis / arthralgias, conjuntivitis, and neurisensorial deafness with hereditary pattern and early onset (<1y in 50% of cases). In last decade, atypical manifestations have emerged expanding the clinical spectrum of CAPS.

## Question

To evaluate the clinical, laboratory and genetic profile of 26 patients of 4 spanish unrelated pedigrees with special attention to the atypical manifestations of the disease.

## Methods

Review of clinical, analytical and genetical data of a cohort of 26 patients from 4 unrelated non-consanguineous Spanish pedigrees.

## Results

Rash (14/26), arthritis (14/26), and deafness (12/26) were the most common features. Episodic fever accounted in 8/26 patients. 14/26 patients did not present episodic course and disease onset was over 10 years in 17/26 patients. 2/26 patients developed amyloidotic hemorrhagic cystitis and other 2/26 patients explained olfactive disfunction. Amyloidosis was confirmed in 2/26 patients and considered as probable in other 4/26 patients. Acute phase reactants were normal in 7/26 patients. CIAS1 mutations were identified in 23/26 patients (Ala439Thr in 5, Arg488Lys in 6 and Arg260Trp in 12). 5/6 patients carriers of an heterozigous Arg488Lys mutation were asymptomatic while it failed to isolate any CIAS1 germinal, somatic or mosaic mutation in 3 members of the same pedigree although their clinical profiles were consistent with Muckle-Wells syndrome (1 case) and CINCA / NOMID (2 cases).

## Conclusions

Despite the traditional clinical picture of CAPS includes periodic fever, rash, arthritis and deafness with onset usually <1 year, in its absence CAPS cannot be ruled out. The clinical spectre may vary from the chronic afebrile course, to atypical amyloidosis, or olfactive disfunction. The profile of CIAS1 mutations carriers may include asymptomatic individuals or absence of mutations in pedigrees with several members with a highly suggestive clinical profile of severe variants of CAPS syndrome.

